# Association of single nucleotide polymorphism rs3213119 variant of *IL-12B* gene in diagnosed Rheumatoid Arthritis patients

**DOI:** 10.12669/pjms.40.5.7671

**Published:** 2024

**Authors:** Iffat Mushtaq, Amir Rashid, Amer Fakhr, Asifa Majeed, Ali Rathore, Zunaira Ali Baig

**Affiliations:** 1 Iffat Mushtaq, MBBS Department of Biochemistry and Molecular Biology, Army Medical College, National University of Medical Sciences, Rawalpindi, Pakistan; 2 Amir Rashid, PhD Department of Biochemistry and Molecular Biology, Army Medical College, National University of Medical Sciences, Rawalpindi, Pakistan; 3 Amer Fakhr, FCPS Department of Rheumatology, Pak Emirates, Military Hospital, Rawalpindi, Pakistan; 4 Asifa Majeed, Post Doc Department of Biochemistry and Molecular Biology, Army Medical College, National University of Medical Sciences, Rawalpindi, Pakistan; 5 Ali Rathore, FCPS Armed Forces Institute of Transfusion, Rawalpindi, Pakistan; 6 Zunaira Ali Baig, MPhil Department of Biochemistry and Molecular Biology, Army Medical College, National University of Medical Sciences, Rawalpindi, Pakistan

**Keywords:** rs3213119, *IL-12B* gene, Polymorphism, Arthritis, Rheumatoid

## Abstract

**Objective:**

To identify the *IL12B* gene variant (rs3213119) and to find its association in Pakistani clinical population of Rheumatoid Arthritis.

**Methods:**

It was a population association (unrelated) case control study, performed from January - December 2022 at Laboratory of Department of Biochemistry and Molecular Biology, Army Medical College, Rawalpindi. Blood samples were collected from all 150 study participants, followed by DNA extraction and Allele-specific polymerase chain reaction performed at Center for Research in Experimental and Applied Medicine (CREAM) Laboratory of Department of Biochemistry and Molecular Biology, Army Medical College Rawalpindi. Statistical analysis was done using ‘SPSS’ (version-22), followed by gene analysis on ‘SNPstat’.

**Results:**

About 28.0% of RA patients were smokers, 38.7% had history of RA in a first degree relative and 70.7% had positive history of consanguinity. Considering rs3213119 variant of *IL12B* gene, frequency of major allele C was 100%, minor allele A was 21%, genotype C/C was 79% and C/A was 21%. Applying the log additive model, the odds ratio of the genotype C/C was 1.00 (adjusted by age and gender with 95 % CI) and the odds ratio of the genotype C/A was 0.00, 52.0% of RA patients originated from four predominant ethnic groups, namely Awaans (18.7%), Rajputs (14.7%), Pathans (12.0%) and Araeens (6.7%).

**Conclusion:**

The study findings suggest the role of minor allele ‘A’ as risk allele in our clinical population. CA genotype confers susceptibility towards the RA development.

## INTRODUCTION

Rheumatoid arthritis (RA) is a recurring auto-immuno-inflammatory disease recognized by advancing joint inflammation and systemic disorders. The key symptoms are pain and swelling of bilaterally affected joints with decreased mobility and can reduce the life span by 10 years with comorbidity to cardiovascular events.^1^ To date, the estimated global prevalence of RA is approximately 1%.^2^ The prevalence of disease is (0.69%) in North America, (0.60%) in Europe (0.34%) in Asia. However, with reference to countries, Finland has the highest prevalence (1.90%) among European states, and (0.90%) in Poland, (0.17%) in the Philippines, 0.7% in India^2^ and 0.142% in Pakistan (Karachi). Women have been reported to be three times more vulnerable and suffer from symptomatic disease between 40-50 years of age.^3^ Certain environmental and genetic factors play a role in the disease onset. Environmental driven genetic predisposition to RA counts for 50-60% of the disease risk.^4^

The data from GWAS has revealed contribution of multiple gene loci and factors to this immune-mediated disease course. A study exhibited 82 risk genes (*BTNL2, HLA-DRA, ATF6B, TAP2, MICAM TRIM31*) associated with the four autoimmune diseases, along with identification of genetic loci related to RA.^5^ A Chinese-based study revealed association of RNA modified SNPs with the RA (*PADI2*, *TRAF1*, *HLA-A*, *HLA-DRB1*, *HLA-DPB1* **and** *HLA-B)*.^6^

Cytokines, as crucial immune cells, play a significant role in various autoimmune diseases. Since cytokines are pivotal in regulating the immune cells, dysfunctional cytokines have been considered to play a significant role in the pathogenesis of autoimmune disease. Interleukins are a family of cytokine which are major immune cells secreted by B cells, and induce the activation and differentiation of immune cells, and play roles in the immune cells’ proliferation, maturation, movement, and adhesion. Approximately 40-50 ILs have been identified so far encoded by the human genome.

Among these interleukins, IL-12 family of cytokines has been found to be associated with disease development.^7^ These interleukins are secreted by monocytes and target T cells and induce T-helper cells 1 (Th1). It also acts as a potent inducer of interferon gamma production by T lymphocytes and NK cells.IL12 is a hetero-dimeric pro-inflammatory cytokine comprised of two subunits; a 35 K-Da subunit coded by the *IL-12A* gene, and a 40 K-Da subunit coded by the *IL-12B* gene. The association of *IL-12B* gene variant (rs3213119) with autoimmune disorders like RA, Psoriasis, Type I DM, Multiple Sclerosis, and Crohn’s disease have been reported.^8^

The *IL12B* gene polymorphism presents a strong genetic predisposing factor to RA while studies establishing association of intronic SNPs of *IL12B* gen with RA have been conducted in Chinese (rs2243115, rs3212227),^9^ Egyptian (rs3212227),^10^ Bulgarian (rs17860508, rs3212227),^11^ and Polish population (rs3212227, rs17860508).^12^ There has not been much research on the *IL12B* gene variants in Pakistan with reference to RA. A little data is available with molecular profiling of the interleukin genes with respect to other diseases.^13^,^14^ The study aimed to explore genetic insight of the *IL12B* variant (rs3213119). This was the first of its kind to probe such an association with RA in the local population.

## METHODS

This population association (unrelated) case control study was conducted from January - December 2022 at the center for research and applied medicine (CREAM) Laboratory of Department of Biochemistry and Molecular Biology, Army Medical College Rawalpindi, in collaboration with Rheumatology Department, PEMH, Rawalpindi.

### Ethical Approval:

A formal approval from the Ethical Review Committee of Army Medical College was taken before commencement of the study (ID#177, dated 19 Jan 2022).

Total sample size was 150, calculated by WHO sample size calculator with an anticipated population proportion of 1%, confidence level of 95% and absolute precision of 0.05. Participants were selected using nonprobability purposive sampling technique. The control group comprised 75 randomly selected healthy individuals (age-matched; both genders) from Armed Forces Institute of Transfusion (AFIT). The participants with status of any other health condition and with RA were excluded while only healthy study subjects were enrolled. The case group comprised 75 individuals with diagnosed RA. The disease severity and activity were assessed and calculated by DAS28, considering the assessments of tenderness and/or swelling of 28 joints, the erythrocyte sedimentation rate (ESR), and patients’ global assessment of their health on a 10 cm visual analogue scale (VAS). DAS Calculator was used to obtain the disease activity values.^15^,^16^ The scoring for determining the disease activity was adopted as; DAS28 ≥2.6 and ≤3.2, moderate disease activity: DAS28 >3.2 and ≤5.1, and high disease activity: DAS28 >5.1 as referenced values. The subjects with any other comorbidity were excluded. Five ml venous blood samples were taken from all study participants, under aseptic measures, after getting their informed written consent.

### Molecular Analysis:

DNA was extracted from blood samples of all recruited study subjects using commercially available kit (FavorPrep Blood Genomic DNA Extraction Mini Kit) followed by gel electrophoresis using 1% agarose, to confirm the presence and quality of extracted DNA. The Information for the gene of interest containing the variant to be studied was obtained from Online Mendelian Inheritance in Man (OMIM) (https://www.omim.org/entry/161561). Sequence of exon (7) of *IL12B* was downloaded from National Center for Biotechnology Information (https://www.ncbi.nlm.nih.gov/nuccore/NG_009618.1). Primers for the rs3213119 variant were designed using online bioinformatics tool “Web-based Allele-Specific PCR assay designing tool (WASP)” available at (https://bioinfo.biotec.or.th/WASP/home). The sequence for wildtype forward primer was 5’-GACAAGACCTCAGCCACAG -3’, for mutant forward primer 5’-GACAAGACCTCAGCCACAT -3’ and for common reverse primer 5’-CTTTCCTCTCCAACACAGC -3’ with product size of 204bp.

Allele-Specific Polymerase Chain Reaction (ARMS-PCR) was carried out to detect the presence of alternate allele of variant. The PCR was carried out in two subsequent reactions: the first involved wildtype forward primer and the second involved mutant forward primer. A common reverse primer was used in both reactions. The ARMS-PCR was carried out following hot start at 95°C for five minutes, thirty-five cycles of denaturation at 95°C for 30 seconds, annealing at 57.2°C for 30 seconds, extension at 72°C for 30 seconds, and final extension at 72°C for seven minutes. The PCR products were resolved on 2% agarose gel to detect the wild/mutant alleles.

### Statistical Analysis:

Clinical parameters were analyzed using SPSS version 22. Categorical data was exhibited as frequency and percentages. Age and other biochemical parameters were expressed as mean ± standard deviation. The two genetic variants were analyzed using online bioinformatics tool SNPstat (https://www.snpstats.net/start.htm). Odds ratio (OR) was used to determine the association of the variants with the disease. To test for Hardy-Weinberg Equilibrium, Exact test was applied, and *p* value < 0.05 was considered statistically significant.

## RESULTS

A comprehensive comparison of demographic and biochemical data of control and case groups is presented in Table-I. The study subjects were divided into two groups, there were more males 37 (49.3%) in the control group as compared to disease group 27 (36%). While there were more females 48 (64%) in the disease group as compared to the control group 38 (50.7%). The maximum number of RA cases were from the region of Punjab (56; 74.7%), followed by KPK (14; 18.7%) and then AJK (6; 8.0%). 39 (52.0 %) cases belonged to four predominant ethnic groups from Punjab, namely Awaans (14; 18.7%), followed by Rajputs (11; 14.7%), Pashto speaking residing in Punjab (9; 12.0%) and then Araeens (5; 6.7%). The polymorphism (rs3213119) is a biallelic variant, having a major allele “C” and a minor allele “A”. Table-II and Table-III illustrate the allelic and genotypic frequencies of both groups. The log additive model was adopted to find association of the rs3213119 variant with RA. It was shown that, with AIC of 148.8 and BIC of 275.2, the homozygous C/C genotype was in 75 (100%) of the controls and 43 (57.3%) of the RA patients, whereas heterozygous C/A genotype was in 0 (0.0 %) of the controls and 32 (42.7%) of RA patients.

**Table-I:** Comparison of Demographic and Biochemical parameters in Control and RA groups.

Parameters Frequency		Controls (N = 75)			RA (N = 75)			p-value

		%	Mean±SD	Frequency	%	Mean±SD		
Gender	Males	37	49.3	---	27	36.0	---	
	Females	38	50.7	---	48	64.0	---	---
Smoking		12	16.0	---	21	28.0	---	0.076
+ve Family History of RA		5	6.7	---	29	38.7	---	<0.001
+ve History of Consanguinity		29	38.7	---	53	70.7	---	<0.001
Age (Years)		---	---	32 ± 7	---	---	46 ± 11	---
ESR (mm/hour)		---	---	12±3	---	---	34±24	<0.001
CRP-Q (mg/L)		---	---	5±2			16±27	<0.001

*p-value < 0.05 as statistically significant.

**Table-II:** Allele and genotype frequencies and SNP Exact test for Hardy-Weinberg equilibrium for the variant rs3213119 of *IL12B* gene.

	Genotypic Frequency			Allele Frequency	

Groups	CC Wild type (homozygous)	CA (heterozygous)	AA Mutant (homozygous)	C	A
Controls	100%	0.0%	0.0%	100%	0.0%
RA group	57%	43%	0.0%	100%	21%

***SNP exact test for Hardy-Weinberg equilibrium (N=150)***					

					*p-value*

All subjects					0.38
Controls					1
RA group					0.032

*p-value < 0.05 as statistically significant.

**Table-III:** Log Additive model used to study the association of variant rs3213119 of *IL12B* gene with RA.

Association of rs3213119 of IL12B gene with RA (N = 150) (Adjusted by Gender + Age)							

Model	Genotype	Controls	RA patients	OR (95% CI)	p-value	AIC	BIC
Log Additive	C/C	75 (100%)	43 (57.3%)	1.00	<0.0001	148.8	275.2
	C/A	0 (0%)	32 (42.7%)	NA (0.00 – NA)			

(OR= Odds Ratio, CI= Confidence interval) (p-value=< 0.05 statistically significant).

The odds ratio of the homozygous genotype (C/C) was 1.00 (adjusted by age and sex with 95 % CI). The odds ratio of the heterozygous genotype (C/A) was insignificant. The Exact test for Hardy-Weinberg equilibrium revealed a *p*-value of one for controls and a *p*-value of 0.032 for RA patients. Increased proportion of consanguineous marriages and positive family history of the disease were detected as important risk factors in the predominant castes as shown in Table-IV.

**Table-IV:** Predominant castes of RA patients with Genotype and Allele frequencies, positive Family History of RA in first degree relative and positive History of Consanguinity.

Pre-dominant Castes	RA (N = 75)	CC Geno- type	CA Geno- Type	C Allele	A Allele	Positive Family History of RA in first degree relative	Positive History of Consanguinity
Awaan	14(18.7%)	8(57.1%)	6(42.3%)	14(100.0%)	6(42.3%)	6(42.8%)	10(71.4%)
Rajput	11(14.7%)	5(45.4%)	6(54.5%)	11(100.0%)	6(54.5%)	5(45.4%)	11(100.0%)
Pathan	9(12.0%)	7(77.8%)	2(22.2%)	9(100.0%)	2(22.2%)	3(33.3%)	7(77.7%)
Araeen	5(6.7%)	2(40.0%)	3(60.0%)	5(100.0%)	3(60.0%)	0(0.0%)	4(80.0%)
Bhatti	3(4.0%)	3(100.0%)	0(0.0%)	3(100.0%)	0(0.0%)	2(66.6%)	2(66.6%)
Malik	3(4.0%)	2(66.6%)	1(33.3%)	3(100.0%)	1(33.3%)	1(33.3%)	2(66.6%)
Mughal	3(4.0%)	2(66.6%)	1(33.3%)	3(100.0%)	1(33.3%)	3(100.0%)	2(66.6%)
Sudhan	3(4.0%)	1(33.3%)	2(66.6%)	3(100.0%)	2(66.6%)	0(0.0%)	2(66.6%)
Chaudhary	2(2.7%)	2(100.0%)	0(0.0%)	2(100.0%)	0(0.0%)	1(50,0%)	1(50.0%)
Jutt	2(2.7%)	1(50.0%)	1(50.0%)	2(100.0%)	1(50.0%)	0(0.0%)	1(50.0%)

## DISCUSSION

RA is an immune-mediated inflammatory disease, characterized by swelling of small joints in feet, hands, wrists and knees. This multifactorial and complex disease requires clinical management strategies, based on environmental factors (lifestyle modifications, diet etc.) and investigation of genetic drivers involved in the disease pathogenesis. A strong family history of the disease marks a significant determinant based on genes involvement. Thus, genetic drivers are strongly influenced by the environment.

The current research study was aimed to explore the presence of the gene variant (rs3213119) in the diagnosed RA patients in our local population. The study manifested the role of gene variant *(IL-12B)* in the risk of disease onset. Various socio demographic factors were considered, to find any possible association with the disease phenotypes, as well. The study presented occurrence of disease in 28.0% of study subjects those who were smokers, while considering the family history as one of the risk factors for the onset of disease; 38.7% study participants showed disease with family history, and 70.7% cases were attributed to consanguinity with first degree relatives. A healthy lifestyle and modified adoptions in habits help reduce the incidence of RA. A study reported association of healthy lifestyle with low risk of RA among female population^17^. Smoking is one of the risk factors which constitute pathological manifestations. Our study reports a bit higher frequency (28%) of the smokers in the RA group compared to that of Control group (16%). There was no statistical difference (*p* value=0.076) found between the two groups with this regard. These findings are in accordance with the local study.^18^

In accordance with family history association with RA in our study, a retrospective study conducted in Hispanic population showed high risk of developing further complications with disease progression having positive family history.^19^ Similarly, research revealed more polygenic risk (10%) with first degree family history in the disease prevalence including RA.^20^ Our study shows the higher number of consanguinity among cases (70.7%), which marks it one of the significant positive factors for the disease occurrence. The prevalence of intermarriages differs in different parts of world but is the most common in Africa, Middle East, and South Asia. The resulting disturbance in pedigree increases the chance of transmitting gene related disorders and manifestation of recessive aberrations.^21^ The overall prevalence of consanguineous marriages n Pakistan is 60% which increases to a disturbing level of 70% in the rural areas of Punjab and KPK and within certain castes like Rajputs and Awaans.^22^ In a Saudi based study, genetic analysis of a first offspring from a first-cousin marriage revealed insertion and deletion of 287-bp Alu sequence in Intron 16 of *ACE* gene.^23^ The pattern of gene inheritance with respect to disease onset often determines the transfer of a particular trait in their subsequent generation. In another study, consanguinity was not significantly associated to susceptibility to T1DM among children.^24^

Our study exhibited statistically higher homozygous genotype (CC) (100%) in control group to that of cases. While the heterozygous genotype CA was only observed in the RA patient group in the adopted model. The presence of alternate allele A among the patients determines the risk of offspring affected with the disease with a strong positive history and consanguinity. However, in a study of Polish population reported significantly higher frequency of minor allele (p=0.037, p=0.04) in the RA group as compared to the control group.^12^

Similarly, in contrast to our findings homozygous (GG and CC) genotypes of rs2243115 and rs3212227 respectively showed a significant association to RA.^9^ The findings from Iraqi population have shown a higher risk of *IL12B* polymorphism (rs32112227) with statistically higher homozygous GG genotype in the patients as compared to the controls.^25^ In any given population, HWE is maintained by certain factors including random mating, non-overlapping generations, insignificant mutation and migration, equal occurrence of allele frequencies in both the sexes, and lack of natural selection.^26^ When any of the above-mentioned factors are violated, then genetic disequilibrium ensues. African/African and American populations demonstrate HWD associated with autosomal recessive diseases due to natural selection, existence of null alleles, segmental duplications, and tandem repeats. The South Asian and Latino/Admixed American populations exhibit HWD due to non-random mating.^27^

In our study, the HWE *p*-value of RA group is 0.032 (<0.05) which indicates a state of HWD due to very high prevalence of consanguinity in the community which coincides with previous reported data. Gene models (co-dominant, dominant, over dominant, recessive, or additive) allow analysis of genotype–phenotype interactions and increase the statistical power of the study to detect disease susceptibility loci.^28^ An additive model assumes that the number of copies of recessive alleles determines the appearance of a discernable trait, more the number of recessive alleles, more intense is the expression of the associated characteristic.^29^

The *IL12B* gene is an autosomal recessive gene and as per log additive model, the expression of minor allele ‘A’ in the diseased group only, might be a risk allele in the off springs of diseased heterozygous (CA) genotype carriers in homozygous pattern of inheritance with positive history of consanguinity and positive family history of RA in first degree relatives as important risk factors. Since a limited data is available on the cytokine related genes and its association in Pakistan, although various global populations have carried out research studies on the respective genes marking its significantly association, it is an emerging need in today’s world to explore the genetic landscape of the disease. Our study is the first study conducted on this variant, to find its possible association with the disease onset and progression, since human’s genetic makeup differs only 0.1% to each other, this difference marks the significance with regards to susceptibility of the disease. Our findings add genetic information on molecular profiling of this gene.

This can be helpful for pharmacogenomics with reference to our population, to target personalized medicine in future. Genetic screening programs for the population at risk of developing RA through identifying genetic analysis of the genes involved may provide ways to improve clinical measures. Further the in-depth understanding of the disease epidemiological parameters can help researchers in the field to devise potential therapeutic strategies.

### Limitations:

The gene variant explored in the current study was not found in the literature of global populations, which may limit generalizing the outcome of the studied polymorphism. The sample size is another limitation of the study, as well as the financial constraints in exploring the molecular architecture of the *IL-12B* gene.

## CONCLUSION

The study findings suggest the role of minor allele ‘A’ as risk allele in our clinical population. The heterozygous (CA) genotype confers susceptibility towards the RA development.

### Authors’ Contribution:

**IM:** did data collection, wet lab work, statistical analysis, manuscript writing and final approval of the manuscript.

**AR** and **AM:** Provided technical assistance, did study design, data interpretation, data review for intellectual content and final approval of the manuscript.

**AF** and **AR:** Helped in data collection.

**ZAB:** Helped in wet lab work and final approval of manuscript.

All authors bear responsibility and accountability for the integrity of the work.
